# Efficacy and safety of the first-line systemic treatments in patients with advanced-stage urothelial carcinoma: a systematic review and network meta-analysis

**DOI:** 10.3389/fonc.2024.1468784

**Published:** 2024-09-16

**Authors:** Yang Zhao, Xiaoqing Xu, Yuhan Sun, Xinyang Yu, Yuanfu Qi, Xin Dai

**Affiliations:** ^1^ First Clinical Medical College, Shandong University of Traditional Chinese Medicine, Jinan, Shandong, China; ^2^ Department of Medical Oncology, Affiliated Hospital of Shandong University of Traditional Chinese Medicine, Jinan, Shandong, China

**Keywords:** advanced-stage, urothelial carcinoma, network meta-analysis, first-line treatments, immunotherapy, antibody-drug conjugates

## Abstract

**Introduction:**

In recent years, some clinical studies of first-line treatment for advanced-stage urothelial carcinoma (aUC) have reached the main endpoint, showing inconsistent clinical efficacy. We hope to explore the efficacy and safety of first-line treatment for aUC.

**Methods:**

The relevant literature from January 2000 to February 2024 was searched, and the R language (version 4.3.1) was used to perform a network meta-analysis based on the JAGS package and GEMTC package under the Bayesian framework. The main indicators included OS, PFS, ORR and adverse events of grade 3 or higher. This study has been registered in PROSPERO (CRD42024525372).

**Results:**

A total of 8 RCTs involving 5539 patients and 12 treatments were included. Pembrolizumab plus Enfortumab Vedotin (PEM+EV) was significantly better than other groups in OS, PFS and ORR. In terms of OS, PEM+EV was significantly better than nivolumab plus platinum-based chemotherapy (NIVO+platinumCT) (HR=0.60; 95% CI: 0.45-0.81), PEM+platinumCT (HR=0.55; 95%CI: 0.42-0.72), atezolizumab (ATE) + platinumCT (HR=0.57; 95%CI: 0.43-0.75) and platinumCT (HR=0.47; 95%CI: 0.38-0.58). In terms of PFS, PEM+EV was also significantly better than NIVO+platinumCT (HR=0.62; 95%CI: 0.48-0.82), PEM+platinumCT (HR=0.58; 95%CI: 0.45-0.74), ATE+platinumCT (HR=0.55; 95%CI: 0.43-0.69) and platinumCT (HR=0.45; 95%CI: 0.38-0.54). In terms of ORR, PEM+EV had a significant be nefit compared with other treatment measures, which was 2.63 times that of platinumCT (OR=2.63; 95%CI: 2.00-3.45). The adverse events of grade 3 or higher in immunotherapy (ATE, PEM, durvalumab) was significantly lower than other treatment measures.

**Conclusions:**

PEM+EV can significantly prolong OS and PFS compared with other treatments, and has a higher ORR. The adverse events of grade 3 or higher of ATE was the lowest.

**Systematic review registration:**

https://www.crd.york.ac.uk/prospero/display_record.php?ID=CRD42024525372, identifier CRD42024525372.

## 1 Introduction

Urothelial carcinoma (UC) is one of the most common malignant tumors of the urinary system, ranking 10th in the incidence of tumors in the world (1). The most common presentation symptoms are hematuria in almost 80% of cases and flank pain in 20% (2). The prognosis of patients with advanced-stage urothelial carcinoma (aUC) is poor, and the 5-year survival rate is only 10% (3). In the 1990s, a phase III study compared the efficacy and safety of GP (gemcitabine+cisplatin) and standard MVAC (methotrexate+vincristine+adriamycincisplatin) in aUC. This study confirmed that there was no significant difference in overall survival (OS) between the two regimens [15.2 months VS 14 months; hazard ratio (HR)=1.09; 95% credible incidence (CI): 0.88-1.34; p=0.66] and the incidence of adverse events (AEs) was lower with GP (4). For patients with cisplatin intolerance, according to the results of the EORTC-30986 study, gemcitabine+carboplatin (GC) was used as a standard chemotherapy regimen for such populations (5). Based on the above research, platinum-based chemotherapy (platinumCT) is the current standard first-line regimen for aUC. The progression-free survival (PFS) of first-line chemotherapy for advanced urothelial carcinoma is about 7.7 months, and the OS is about 10-15 months (4). However, about 30%-50% of aUC patients cannot tolerate platinumCT due to advanced age or combined underlying diseases/Eastern Cooperative Oncology Group Performance Status (ECOG PS)≥3 (6). Clinically, the first-line treatment of aUC has been under exploration. In recent years, with the breakthrough of immune-checkpoint inhibitors (ICIs) represented by programmed death-1 (PD-1) and programmed death-ligand 1 (PD-L1) inhibitors, and gradually moving to the first-line treatment and maintenance treatment, the treatment of aUC has also advanced from single chemotherapy to combined treatment. ICIs become a new choice for patients with platinum intolerance. Consensus exists between the American Urologist Association (AUA), European Association of Urology (EAU), and the National Comprehensive Cancer Network (NCCN) in supporting the use of adjuvant ICIs in patients with high-risk muscle-invasive UC who had undergone radical surgery (2). Since May 2016, atezolizumab (ATE), nivolumab (NIVO), avelumab, and pembrolizumab (PEM) have been approved by the Food and Drug Administration for first-line or second-line treatment of aUC and related clinical trials. The IMvigor130 study and the KEYNOTE-361 study evaluated the efficacy of ATE and PEM alone or in combination with chemotherapy as first-line treatment for aUC, respectively. Unfortunately, the results showed that compared with standard chemotherapy, both ATE and PEM did not improve the OS (7, 8). In the Phase III CheckMate-901 study, both PFS and OS achieved positive results. The results showed that the OS of NIVO+platinumCT group was 21.7 months, while the platinumCT group was 18.9 months (HR=0.78; 95%CI: 0.63-0.96; p=0.0171); In terms of PFS, the combined group was 7.9 months, and the platinumCT group was 7.6 months (HR=0.72; 95%CI:0.59-0.88; p=0.0012) (9). It is worth noting that the median complete remission time of the combined group was 37.1 months, almost three times the 13.2 months of the CT group. This study confirmed for the first time that immune combined with chemotherapy can bring long-term efficacy benefits to aUC patients. In recent years, antibody-conjugated drugs (ADC) have been actively explored as a new anti-tumor strategy in the field of multiple solid tumors. ADC combines the specificity of monoclonal antibodies and the cytotoxicity of chemotherapeutic drugs to achieve a precise strike on tumor cells (10). Among them, Enfortumab Vedotin (EV) as a representative of ADC, has carried out a series of clinical studies in UC. This drug uses Nectin-4 as a target, and the anti-Nectin-4 human immunoglobulin G1 antibody Enfortomab is coupled with the microtubule-destroying agent methylreositine E (MMAE) through an enzymatic linker containing valine-citrulline and the drug-antibody ratio is 3.8 (11). The EV-301 study evaluated the efficacy of EV versus platinumCT in the previously treated patients, which showed that the OS of the EV was prolonged by 3.91 months (12.88 months VS 8.97 months; HR=0.7; 95%CI:0.56-0.89; p=0.00142) (12). The EV-302 study was designed to evaluate the efficacy and safety of pembrolizumab and Enfortumab Vedotin (PEM+EV) compared with platinumCT in the first-line treatment of aUC. The latest data showed that the OS (31.5 months VS 16.1 months; HR=0.47; 95%CI:0.38-0.58; p<0.00001) and PFS (12.5 months VS 6.3 months; HR=0.45; 95%CI:0.38-0.54; p=0.00001) of the PEM+EV were significantly prolonged (13). This study provides a higher level of evidence for PEM+EV as a first-line treatment for aUC, breaking the clinical dilemma of limited first-line treatment options and poor benefits for aUC.

In summary, there is still a lack of direct or indirect comparison of different treatments. Therefore, we conducted a systematic review and network meta-analysis (NMA) to evaluate the role of different treatment regimens (chemotherapy, targeted therapy, immunotherapy, ADC) in the first-line treatment of aUC and to provide valuable evidence for clinicians to choose the best first-line treatment for patients.

## 2 Materials and methods

### 2.1 Protocol and registration

This NMA was performed by the PRISMA extension statement for NMA (**Supplementary Table 1**). The protocol for this study has been registered in PROSPERO (CRD42024525372).

### 2.2 Data sources and search strategy

PubMed, Embase, the Cochrane Central Register of Controlled Trials, and ClinicalTrials.gov databases were searched to find relevant articles from January 2000 to February 2024. Abstracts on UC from several important international conferences (American Society of Clinical Oncology, ASCO; European Society for Medical Oncology, ESMO) from 2000 to 2024 were inspected to identify potentially relevant studies. The detailed search strategy is shown in **Supplementary Table 2**.

### 2.3 Inclusion and exclusion criteria

Inclusion criteria:

1. Randomized controlled trials (RCTs) that enrolled patients with aUC (stages IV) confirmed either histologically or cytologicallly.
2. RCTs that explored the first-line treatment of aUC.
3. RCTs that were published or published in the form of conference abstracts, and reported results such as OS/PFS/Objective Response Rate (ORR)/AEs.

excluded criteria:

1. RCTs that included unclear organizational types.
2. RCTs that discussed preoperative neoadjuvant therapy or conversion therapy.
3. RCTs that included radiotherapy, cytokines, tumor vaccines, or immune cell therapy.
4. RCTs that the main purpose is to study the quality of life or economic benefits.

### 2.4 Data extraction and quality assessment

We extracted the data of ID, first author, year of publication, number of patients and patient characteristics, treatment methods, and results of eligible studies into a spreadsheet. The Cochrane bias risk tool was used to assess the risk of bias in 8 studies (7, 9, 13–19). The tool was based on random sequence generation, allocation concealment, blinding of participants and personnel, blinding of outcome assessment, incomplete outcome data, selective outcome reporting, and other sources of bias to assess the risk of bias in the study. Finally, each study was divided into low, high, or unclear risk of bias (20). Two researchers (YZ and XQX) independently assessed the risk of bias in each trial and independently performed literature screening and data extraction. Any inconsistency shall be resolved by arbitration by the corresponding author.

### 2.5 Statistical analysis

We performed this NMA of 8 RCTs containing control groups according to the PRISMA network meta-analysis extension statement and compared the efficacy and safety of different treatment measures through the HR(PFS and OS) of each treatment measure or the odds ratio (OR) (ORR and ≥3AEs) of the dichotomous results and its corresponding 95% CI. We used the Q test and I2 statistics in NMA to evaluate the heterogeneity between studies in forest plots. Different effect models were selected according to different I2 values and p values. The random effect model was selected for I2 ≥50%, and the fixed effect model was selected for I2<50% (21).

We conducted an NMA of 8 RCTs containing control groups using the R language (version 4.3.1) based on the JAGS package and the GEMTC package under the Bayesian framework. For each calculation result, 150,000 iterations, 100,000 annealings, and 1 step are used. In order to ensure the convergence of the model, the method of diagnostic convergence graph and trajectory density graph is adopted (**Supplementary Figure 1**). To determine the best treatment, we calculated the surface under the cumulative ranking curve (SUCRA). The larger the SUCRA value, the greater the likelihood that a measure will be in the top position. Different effect models were selected according to the difference of DIC values. When the DIC value difference is less than 5, the fixed effect model is selected, and the random effect model is selected when≥5 (22).

## 3 Results

**Figure 1:**
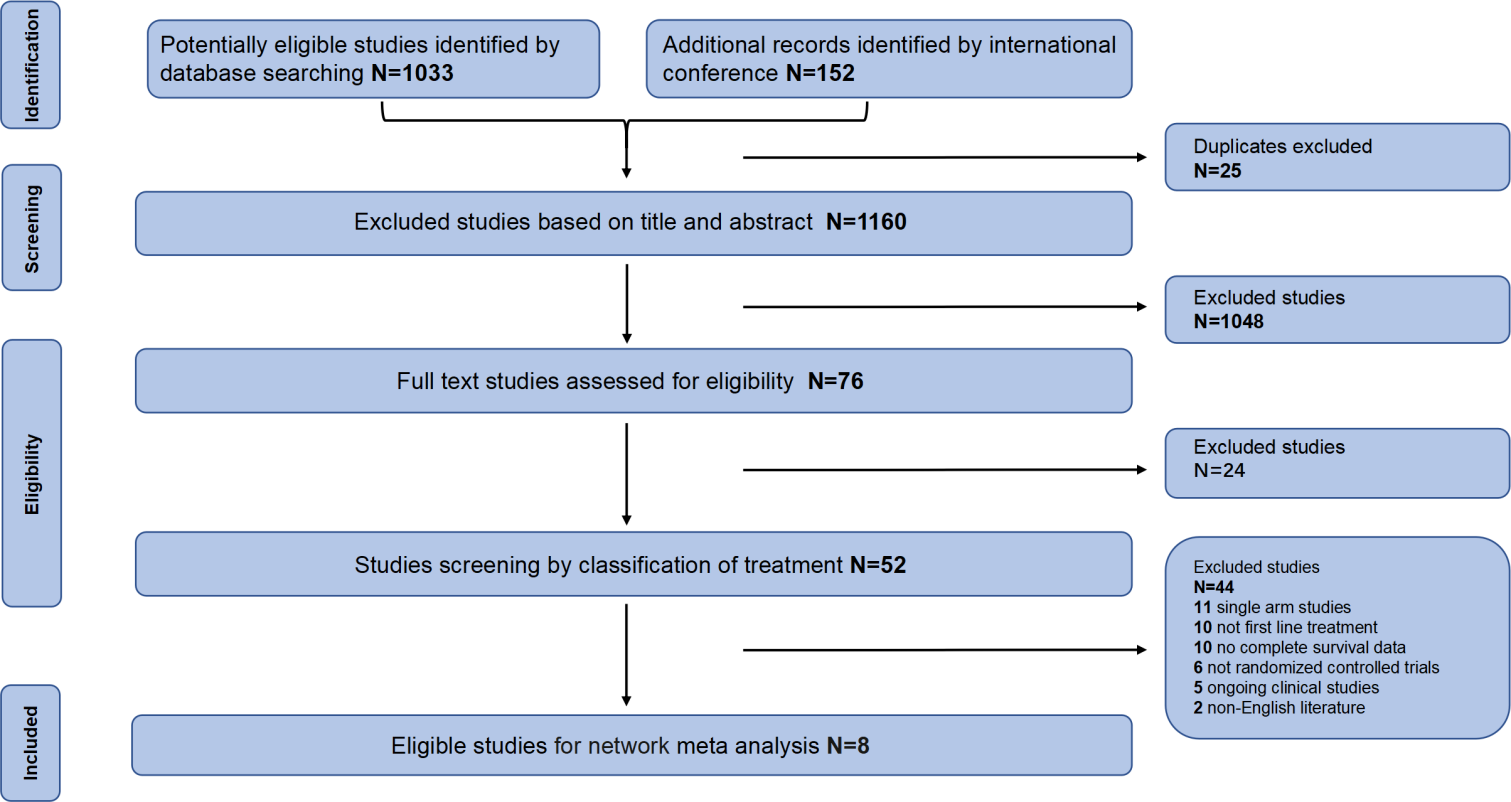
Flow chart of study selection.

**Figure 2:**
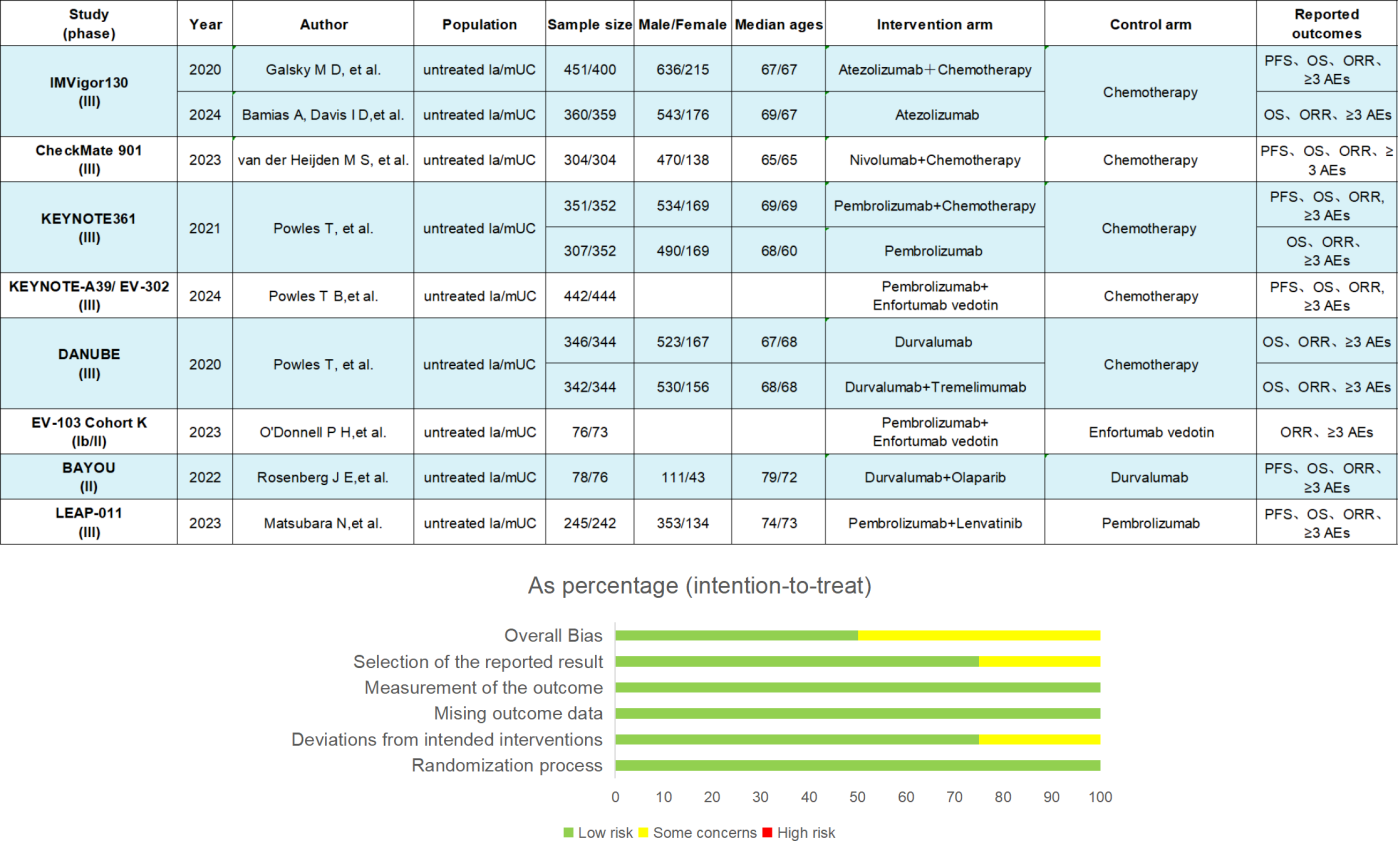
Key study features. The summary of results from bias risk assessment of studies was made from the Cochrane risk of bias tool. la/mUC, locally advanced/metastatic urothelial carcinoma; OS, overall survival; PFS, progression-free survival; ORR, objective response rate; AE, adverse events.

**Figure 3:**
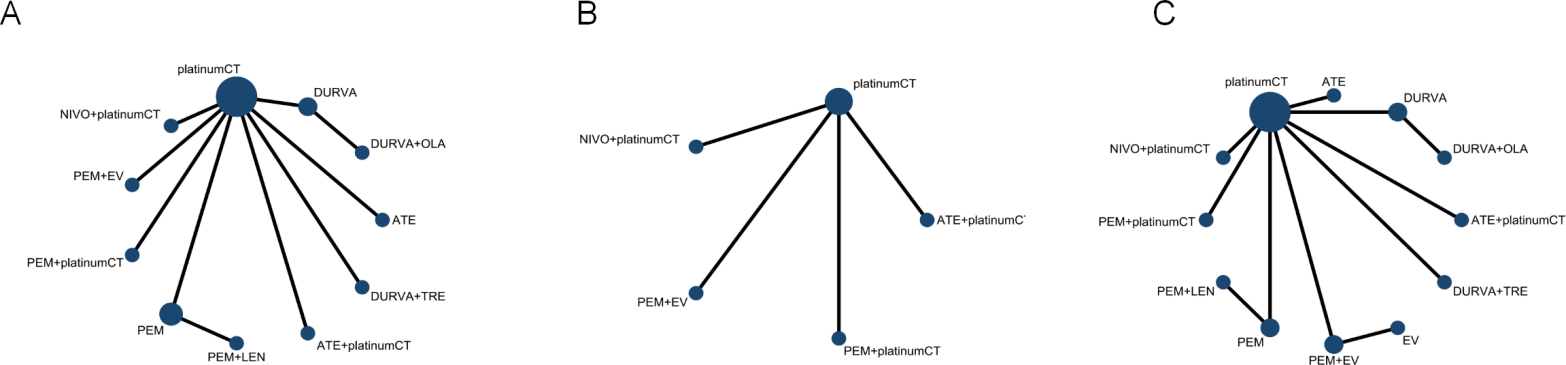
Network diagram comparing different treatments. **(A)** Network diagrams comparing overall survival, **(B)** progression-free survival and **(C)** objective response rate and adverse events. Each circular node represents a type of treatment. Each line represents a type of head-to-head comparison. The size of the nodes and the thickness of the lines are weighted according to the number of studies evaluating each treatment and direct comparison. platinumCT, platinum-based chemotherapy, NIVO+platinumCT, nivolumab plus platinum-based chemotherapy, ATE+platinumCT, atezolizumab plus platinum-based chemotherapy; PEM+platinumCT, pembrolizumab plus platinum-based chemotherapy; ATE, atezolizumab; PEM, pembrolizumab; PEM+EV, pembrolizumab plus enfortumab vedotin; DURVA. Durvalumab; DURVA+TRE, durvalumab plus tremelimumab; DURVA+OLA, durvalumab plus olaparib; PEM+LEN, pembrolizumab plus lenvatinib; EV, enfortumab vedotin.

According to the inclusion and exclusion criteria, we finally identified 8 studies (**Figure 1**) with a total of 5539 patients, including 12 treatment regimens: platinumCT, Nivolumab plus platinum-based chemotherapy (NIVO+platinumCT), Atezolizumab plus platinum-based chemotherapy (ATE+platinumCT), Pembrolizumab plus platinum-based chemotherapy (PEM+platinumCT), ATE, PEM, PEM+EV, EV, Durvalumab (DURVA), Durvalumab plus tremelimumab (DURVA+TRE), Durvalumab plus olaparib (DURVA+OLA), and Pembrolizumab plus lenvatinib (PEM+LEN) (**Figure 2**). Then analyzed and compared the OS (**Figure 3A**), PFS (**Figure 3B**), ORR, and ≥3AEs (**Figure 3C**) of each treatment regimen. In addition, we performed subgroup analysis of age, gender, and PD-L1 expression based on the PRISMA principle (**Supplementary Figure 2**).

### 3.1 OS and PFS

**Figure 4:**
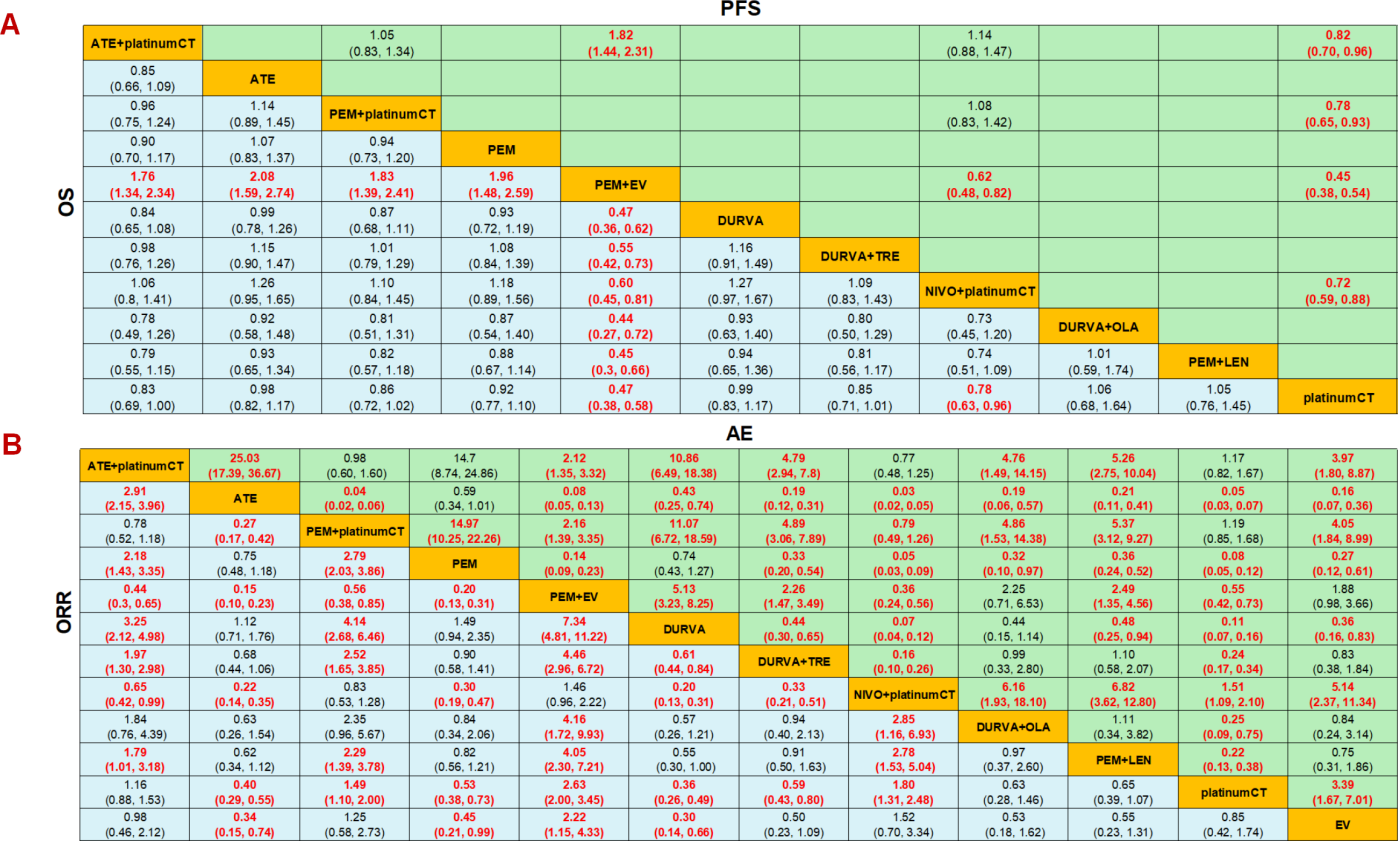
Efficacy and safety profiles of the Bayesian network meta-analysis in advanced-stage urothelial carcinoma. **(A)** Hazard ratios (HR) and 95% credible intervals (95% CI) of OS and PFS. **(B)** Odds ratio and 95% CI of ORR and adverse events of grade 3 or higher. Data in each cell are HR and 95% CI for the comparison of row-defining treatment versus column-defining treatment. HR less than 1 favors upper-row treatment. Significant results are highlighted in red. platinumCT, platinum-based chemotherapy; NIVO+platinumCT, nivolumab plus platinum-based chemotherapy; ATE+platinumCT, atezolizumab plus platinum-based chemotherapy; PEM+platinumCT, pembrolizumab plus platinum-based chemotherapy; ATE, atezolizumab; PEM, pembrolizumab; PEM+EV, pembrolizumab plus enfortumab vedotin; DURVA, durvalumab; DURVA+TRE, durvalumab plus tremelimumab; DURVA+OLA, durvalumab plus olaparib; PEM+LEN, pembrolizumab plus lenvatinib; EV, enfortumab vedotin.

The results showed that the OS and PFS of PEM+EV were significantly longer than those of other measures, and the regimen had significant survival benefits compared with immunotherapy combined chemotherapy or dual-drug immunotherapy. In terms of OS, PEM+EV was significantly better than immune combined chemotherapy including NIVO+platinumCT (HR=0.60; 95%CI: 0.45-0.81), PEM+platinumCT (HR=0.55; 95%CI: 0.42-0.72), ATE+platinumCT (HR=0.57; 95%CI: 0.43-0.75), which was also better than immunotherapy including PEM (HR=0.51; 95%CI: 0.39-0.67), ATE (HR=0.48; 95%CI: 0.36-0.63), DURVA (HR=0.47; 95%CI: 0.36-0.62), DURVA+TRE (HR=0.55; 95%CI: 0.42-0.73), DURVA+OLA (HR=0.44; 95%CI: 0.27-0.72), PEM+LEN (HR=0.45; 95%CI: 0.30-0.66) and current first-line platinumCT (HR=0.47; 95%CI: 0.38-0.58). At the same time, NIVO+platinumCT was significantly better than platinumCT (HR=0.78; 95%CI: 0.63-0.96) (**Figure 4A**). In terms of PFS, PEM+EV was significantly better than immunotherapy combined chemotherapy including NIVO+platinumCT (HR=0.62; 95%CI: 0.48-0.82), PEM+platinumCT (HR=0.58; 95%CI: 0.45-0.74), ATE+platinumCT (HR=0.55; 95%CI: 0.43-0.69) and platinumCT (HR=0.45; 95%CI:0.38-0.54). In addition, immune combined chemotherapy NIVO+platinumCT (HR=0.72; 95%CI: 0.59-0.88) was significantly superior to platinumCT in PFS (**Figure 4A**).

### 3.2 ORR and AEs

In terms of ORR, PEM+EV has a significant benefit compared to other treatment measures, which is 2.63 times that of platinumCT (OR=2.63; 95%CI: 2.00-3.45), and is also significantly better than PEM+platinumCT(OR=1.77; 95%CI: 1.18-2.65) and ATE+platinumCT (OR=2.26; 95%CI: 1.53-3.34), but there is no significant difference compared with NIVO+platinumCT (OR=1.46; 95%CI: 0.96-2.22). PEM+platinumCT (OR=1.49; 95%CI: 1.10-2.00) and NIVO+platinumCT (OR=1.80; 95%CI: 1.31-2.48) achieved higher ORR than platinumCT, but there was no significant difference between ATE+platinumCT and platinumCT (OR=1.16; 95%CI: 0.88-2.53) (**Figure 4B**). In terms of AEs, the incidence of ≥3AEs in immunotherapy (ATE, PEM, DURVA) was significantly lower than other treatment measures, while the incidence of ≥3AEs in immunotherapy combined with chemotherapy (ATE+platinumCT, PEM+platinumCT, NIVO+platinumCT) was significantly higher than chemotherapy. It is worth noting that the serious adverse reactions of PEM+EV were significantly lower than PEM+platinumCT, NIVO+platinumCT and ATE+platinumCT, but significantly higher than platinumCT regimen or EV (**Figure 4B**).

### 3.3 Rankings

**Figure 5:**
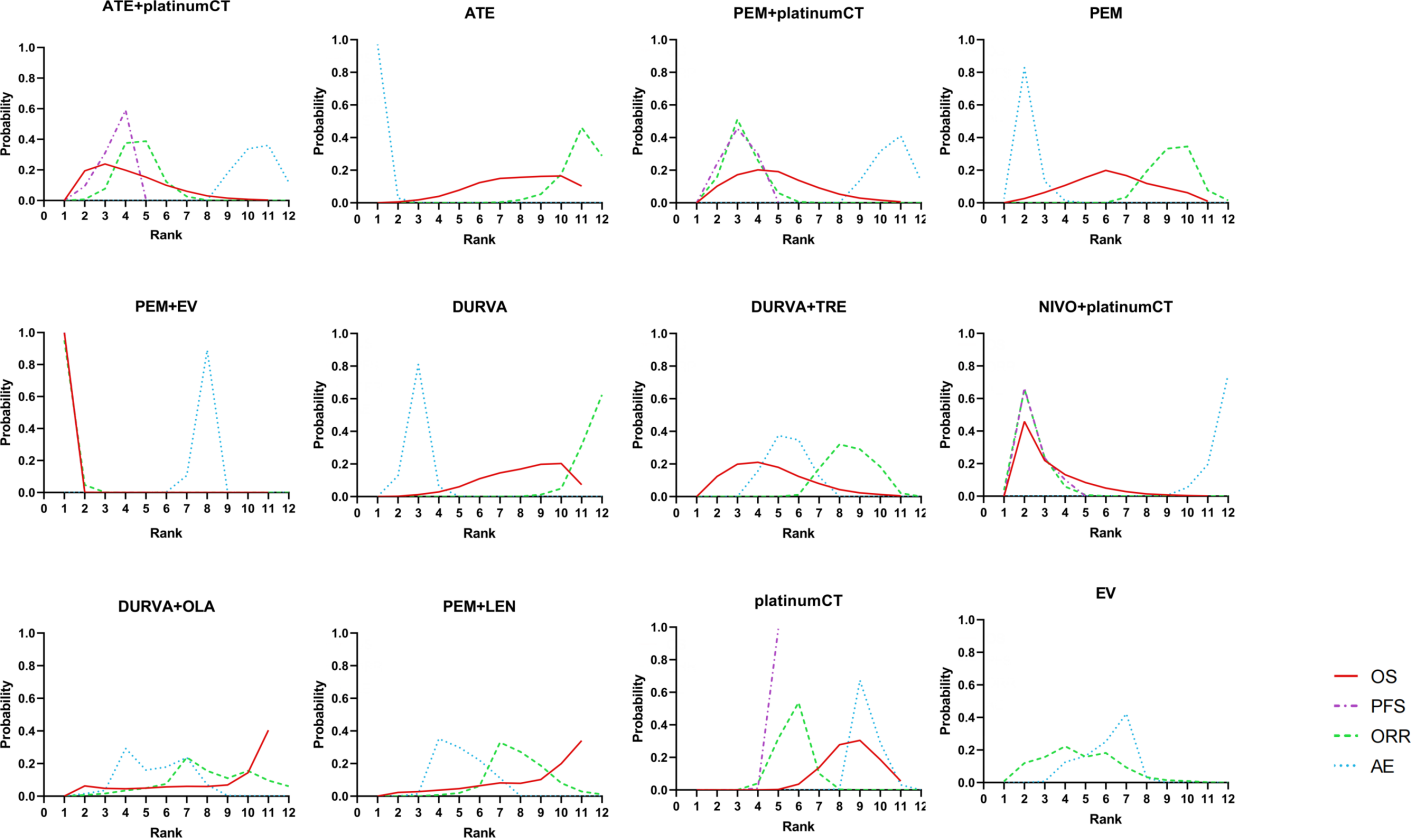
Bayesian ranking profiles comparing efficacy and safety of different treatment. Ranking plots indicate the probability of each comparable treatment strategies being ranked from first to last on overall survival, progression-free survival, objective response rate, and adverse events of grade 3 or higher. platinumCT, platinum-based chemotherapy; NIVO+platinumCT, nivolumab plus platinum-based chemotherapy; ATE+platinumCT, atezolizumab plus platinum-based chemotherapy; PEM+platinumCT, pembrolizumab plus platinum-based chemotherapy; ATE, atezolizumab; PEM, pembrolizumab; PEM+EV, pembrolizumab plus enfortumab vedotin; DURVA, durvalumab; DURVA+TRE, durvalumab plus tremelimumab; DURVA+OLA, durvalumab plus olaparib; PEM+LEN, pembrolizumab plus lenvatinib; EV, enfortumab vedotin.

For patients with aUC, the first-line application of PEM+EV ranked first in OS, PFS, and ORR with cumulative probability of 99.9%, 99.9%, and 99.5%, respectively. The incidence of ≥3AEs of ATE (99.7%) was the lowest (**Figure 5**).

### 3.4 Subgroups

Only IMvigor130 study, CheckMate 901 study, including NIVO + platinumCT, ATE, platinumCT three treatment regimens can extract data and perform subgroup analysis. Patients were divided into the following subgroups according to age (≥ 65 years & < 65 years) (**Supplementary Figure 3A**), gender(male & female) (**Supplementary Figure 3B**) and PD-L1 expression (negative & positive) (**Supplementary Figure 3C**). Subgroup results showed that there was no significant difference in subgroups of age, gender and PD-L1 expression in OS.

## 4 Discussion

As mentioned above, to compare and evaluate the efficacy of first-line regimens for aUC, we conducted a NMA of the outcome indicators of these first-line treatment strategies. The results confirmed that PEM+EV can achieve significant benefits in both short-term PFS, ORR and long-term OS compared with other treatment measures. The incidence of ≥3AEs in immunotherapy was the lowest, while the incidence of PEM+EV was significantly lower than that of immunotherapy combined with chemotherapy (ATE+platinumCT, PEM+platinumCT, and NIVO+platinumCT).

PlatinumCT has been approved for long-term clinical application as a first-line regimen for advanced UC according to the results of EORTC-30986 and other studies since the 1990s (4, 5). However, the efficacy of this regimen is limited, and fails to translate short-term PFS benefits into OS benefits. This has long been a major challenge in the treatment of aUC. Although the immunotherapy combined chemotherapy regimen is widely used in multiple tumors, no positive results were obtained in the IMvigor130 study and the KEYNOTE-361 study (7, 8). The results of our NMA showed that compared with the positive results in the CheckMate-901 study, NIVO+platinumCT also ranked second in the SUCRA ranking with a cumulative probability of 77.5%, after the immunotherapy combined ADC (PEM+EV) (9).

As a new anti-tumor strategy, PEM+EV ranked first in this NMA with absolute advantages in OS, PFS, and ORR data, and the results of the EV-302 study subgroup confirmed that it can benefit regardless of cisplatin tolerance and PD-L1 expression level (13). ADC is a class of anti-tumor drugs that are coupled by a linker to a humanized monoclonal antibody (mAb) targeting a specific antigen and payload composed of cytotoxic small molecule drugs (23, 24). The main pathways of this drug’s anti-tumor activity are: specific mAb bind to targeted cell surface antigens, are internalized by tumor cells and processed by the internal lysosomal system, and small molecule drugs that are effectively loaded are released into the cytoplasm. The anti-tumor activity of ADC is through the binding of specific mAbs to targeted cell surface antigens, which are internalized by tumor cells and processed by the endosomal system. Subsequently, the effective payload is released into the cytoplasm, and finally induces apoptosis through the cytotoxic pathway and bystander effect (10). Based on the results of EV-201 study cohort 1, EV became the first ADC approved for the treatment of UC in 2019 (25). In our NMA, PEM+EV achieved an absolute advantage in anti-tumor efficacy. At the same time, compared with EV-302 study results, PEM+EV significantly prolonged OS and PFS compared with chemotherapy (OS: 31.5 months VS 16.1 months, HR=0.47; 95%CI: 0.38-0.58; p<0.00001, PFS: 12.5 months VS 6.3 months, HR=0.45; 95%CI: 0.38-0.54; p<0.00001) (13). The breakthrough results of this study enabled PEM+EV to successfully challenge the first-line status of platinumCT and become the first approved first-line combination of immunotherapy and ADC for aUC treatment. The reason may be related to the following factors: first, EV targets on the Nectin-4, which promotes tumor cell proliferation, differentiation, metastasis by activating PI3K/AKT pathway, and plays a role in tumor formation (26). High expression of Nectin-4 was found in 60% of UC (27). Compared with chemotherapy, EV has more targeting ability. Secondly, EV has a bystander effect, that the ADC drug is internalized and releases small, uncharged, permeable membrane hydrophobic molecules that diffuse into the cell membrane and kill the tumor cells in the case of negative expression of adjacent antigens (28). Preclinical models have demonstrated that non-targeted ADC is effective in the presence of tumor-associated macrophages. ADC is internalized and processed by the FCɣ receptor expressed by macrophages, releasing a payload in the tumor microenvironment, and then blocking by antibody-mediated receptor signaling to kill adjacent target-negative tumor cells (29). This feature may contribute to ADC activity against tumors with heterogeneous or low target antigen expression. This effect may make EV better act on metastatic tumor tissues and more effective for metastatic UC with high expression of Nectin-4. In addition, the combination of ICIs (anti-PD-1 and anti- Cytotoxic T-lymphocyte-associated antigen 4) is worthy of attention. The NABUCCO study found that the pathological complete response rate of preoperative treatment of ipilimumab + nivolumab in patients with resectable urothelial carcinoma was 46% (30), and the CheckMate 032 study also confirmed the effectiveness of ipilimumab + nivolumab in patients with aUC who had previously received platinum therapy (31). Therefore, we are looking forward to the exploration of the first-line treatment efficacy of ICIs combination in aUC.

It is worth considering that PEM+platinumCT did not show significant survival benefit compared with platinumCT, while PEM+EV showed significant survival benefit compared with PEM+platinumCT, PEM+LEN, and platinumCT, indicating that ADC had a more Immune activation effect than multi-target tyrosine kinase inhibitors (TKI) and traditional chemotherapy. In the study of mouse model, it was also confirmed that the effect of immunotherapy combined with ADC therapy was synergistic, not simply additive (32). This may be due to the induction of immunogenic cell death (ICD) and injury-related molecular patterns (DAMP) to activate dendritic cells to promote tumor and immune cell interactions, and ultimately provide potential synergistic effects for immunotherapy. The death of tumor cells can be immune or non-immune. ICD is a regulated form of cell death, including induction of endoplasmic reticulum and cell stress, accompanied by changes in cell surface composition and release of soluble mediators (33, 34). This cell death pattern includes the “eat me” signal exposed on the cell surface, promoting the absorption of dying cells by phagocytes, and extracellular release of immune-stimulating factors, which promoting anti-tumor immune response (35, 36). Preclinical model studies have confirmed that most cytotoxic payloads for ADC can stimulate immune cells *in vitro* or *in vivo* and enhance the anti-tumor effect of ICIs (37). In the mouse model, it was confirmed that the payload MMAE used by EV itself can induce ICD and promote anti-tumor immune response, while the immune system can reversely enhance the anti-tumor activity of this ADC (38). Mature dendritic cells (DCs) play a pivotal role in cancer immunity due to their role as antigen-presenting cells that can stimulate, via the MHC class II complex, anti-tumor T cell responses (39). Tumor cells can lead to immunosuppressive effects by inhibiting the maturation of DCs or inducing dysfunction and ultimately produce immune escape (40). Overcoming the inhibitory effects of DCs is the key to enhancing the efficacy of immunotherapy. In preclinical models, the payload carried by the ADC was found to induce the activation and maturation of dendritic cells and the production of proinflammatory cytokines (32). This finding suggests that ADC promotes the initiation and expansion of T cells by promoting the antigen uptake of DCs and the migration to tumor-draining lymph nodes, which leads to increased infiltration of CD8+ T cells in the tumor microenvironment, thereby promoting the efficacy of immunotherapy.

NIVO+platinumCT achieved significant PFS and OS benefits compared with platinumCT, while PEM+platinumCT and ATE+platinumCT did not achieve significant survival benefits compared with platinumCT. A careful comparison of the studies showed that 36.5% of the population included in the CheckMate-901 study had positive expression of PD-L1. 45% of the population in the Keynote-361 study had CPS≥10, and 67% of the population in the IMvigor130 study had TC/IC>1. It is suggested that PD-L1 expression may not be a single biomarker for predicting mUC first-line immunotherapy. The results of subgroup analysis also supported this idea. The OS results of PD-L1 (+) subgroup showed that NIVO + platinumCT was higher than platinumCT (HR = 0.75; 95% CI: 0.46-1.22). A meta-analysis showed that clonal tumor mutation burden (TMB), total TMB and APOBEC signature were the most relevant predictive features for the efficacy of immunotherapy in UC (41). This analysis also confirmed that TRAF2 deletion is a predictor of ICIs response, and CCND1 amplification is a marker of immunotherapy resistance in UC. Biomarker analysis data from clinical trials have shown that somatic mutations in DNA damage response or cell cycle regulatory genes are also effective biomarkers for predicting the efficacy of immunotherapy (41, 42). The ctDNA analysis showed a significant correlation between the reduction in the frequency of FGFR changes and superior OS by immunotherapy (43). In summary, the exploration of immunotherapy combined with chemotherapy in aUC first-line treatment still needs to find an appropriate biomarker to predict the efficacy.

In terms of the safety of each treatment measure, the overall AEs and the ≥3AEs of immunotherapy monotherapy (ATE, PEM, NIVO) were significantly lower than other treatment measures. The ≥3AEs of PEM+EV were higher than chemotherapy but lower than immunotherapy combined chemotherapy (ATE+platinumCT, PEM+platinumCT, NIVO+platinumCT). This suggests that PEM+EV was safe and tolerable, and NIVO+platinumCT was found to have the highest AEs through our NMA. Therefore, PEM+EV not only shows significant survival benefits but also is tolerable for aUC patients.

Cost-effectiveness is also an unavoidable topic. Immunotherapy plus ADC increase the economic burden of patients, and cost-effectiveness is crucial for determining the best and most sustainable treatment strategies in the future (44). At present, a phase III study compared the effects of standard-dose immunotherapy and low-dose regimens on patients with different types of tumors (45). The results of this study may provide valuable evidence to support alternative treatment regimens that strike a balance between maintaining therapeutic effects, providing cost-effective treatment, and minimizing treatment-related toxicity, which may ultimately bring better quality of life to patients. Biomarkers, as a personalized treatment option, will provide a basis for the best drug selection of patients.

In conclusion, the treatment regimen of immunotherapy combined with ADC is superior to immunotherapy combined with chemotherapy in terms of efficacy and safety. However, there is currently a lack of head-to-head large-sample phase III RCTs to compare immunotherapy and ADC with other immunotherapy combined regimens. The EV-302 study has initially verified the efficacy of PEM+EV and opened a new era of immunotherapy combined with ADC. At the same time, more immunotherapy combined with ADC regimens are expected to be applied to aUC and other tumors.

There are still some shortcomings in this NMA. First of all, in the included studies, some studies did not provide subgroup analysis and some studies did not have uniform subgroup classification criteria, which made it impossible to compare all treatment methods for each subgroup. Secondly, the LEAP-011 and BAYOU studies included patients with platinum intolerance, which may affect the accuracy of the results. In addition, our study only aimed at patients with common pathological types of UC, and there was no further distinction and exploration of the variant urothelial carcinoma (46). For example, micropapillary, plasmacytoid, small cell and sarcomatoid subtypes appear to be associated with poor survival outcomes, while lymphoepithelioma-like subtypes appear to be better prognosis (47–49).

## 5 Conclusions

Through this NMA, we found that in the first-line treatment of aUC, PEM+EV regimen could significantly prolong OS and PFS compared with other regimens, and has a higher ORR. The Incidence of ≥3AEs withATE was the lowest. The Incidence of ≥3AEs with PEM+EV were higher than chemotherapy but lower than immunotherapy combined with chemotherapy (ATE+platinumCT, PEM+platinumCT, NIVO+platinumCT). This suggests that immune combined with ADC may be the optimal choice in aUC, whether considering efficacy and safety. We hope that these results can provide a more accurate choice for first-line clinical treatment of aUC.

## Data Availability

The raw data supporting the conclusions of this article will be made available by the authors, without undue reservation.
